# Technical-scientific production and knowledge networks about medicinal plants and herbal medicines in the Amazon

**DOI:** 10.3389/frma.2024.1396472

**Published:** 2024-06-12

**Authors:** Nadja Lepsch-Cunha, Vinicius Muraro, Henrique Eduardo Mendonça Nascimento, Alysson Mazoni, Cecília Verónica Nunez, Maria Beatriz Machado Bonacelli

**Affiliations:** ^1^General Coordination of Research, Training and Extension, National Institute of Amazonian Research – INPA, Manaus, AM, Brazil; ^2^Research Policy, Department of Business Administration, School of Economics and Management, Lund University, Lund, Sweden; ^3^Coordination of Biodiversity, National Institute of Amazonian Research – INPA, Manaus, AM, Brazil; ^4^Department of Science and Technology Policy, Institute of Geosciences, Universidade Estadual de Campinas (Unicamp), Campinas, SP, Brazil; ^5^Technology and Innovation Coordination - COTEI, National Institute of Amazonian Research – INPA, Manaus, AM, Brazil

**Keywords:** scientometrics, social network analysis, Technology Readiness Level, Brazil, neglected tropical disease, Amazon traditional medicine, S&T planning

## Abstract

**Introduction:**

This paper explores the role of Brazilian research institutions in the global and national context of study of medicinal plants. Most of these plants have ethnopharmacological use and herbal medicines related to the Amazon. It highlights Brazil's position in scientific production and the importance of Amazonian resources in developing phytomedicines. The study aims to provide an overview of the technical-scientific production of medicinal plants and herbal medicines related to the Amazon, focusing on scientific impact, collaboration, Technology Readiness Level (TRL) of scientific production, and innovation system maturity.

**Methods:**

The study employs a comprehensive methodological approach, including data collection from Scopus covering the period from 2002 to 2022. The data was cleaned and analyzed using bibliometric and network analysis techniques. Advanced natural language processing techniques, such as Latent Dirichlet Allocation and Jaccard distance measure, were used for TRL classification.

**Results:**

The findings reveal a predominant contribution from Brazilian institutions and authors, with 1,850 publications analyzed. Key areas identified include Pharmacology, Toxicology, Pharmaceuticals, Medicine, and Biochemistry. The study also uncovers various collaborative networks and technological maturity levels, with a significant focus on early-stage development phases.

**Discussion:**

The research concludes that Brazilian institutions, particularly those in the Amazon region, play a significant role in the scientific exploration and development of medicinal plants and herbal medicines. Despite this, countries like the USA were proportionally more productive in clinical trial research. The study underscores the potential of Brazil's rich biodiversity and traditional knowledge in the pharmaceutical industry, particularly for neglected diseases. It suggests the need for stronger research systems and international collaboration to leverage these resources for global health benefits.

## 1 Introduction

Although of expressive use by human populations for millennia, the current increasing demand for healthy natural products is not trivial and creates a worldwide need for regulation of these products (WHO, 2013). In response, the Brazilian government enacted the National Policy of Medicinal Plants and Herbal Medicines (PNPMF) in 2006 (Brasil, 2014), establishing a legal framework for their rational use. This policy outlines various actions for research, development, and innovation.

The creation of regulated markets for medicinal plants within the Brazilian healthcare system is a key driver of innovation. This issue attracts both public and private interest, being a crucial component in enhancing the competitiveness of Brazil's healthcare industry (Hasenclever et al., 2017). However, it's essential to further integrate the different stages of the phytotherapeutic production chain, thereby improving collaboration among raw material producers, science and technology institutions, and companies (Guilhermino et al., 2012; Villas-Bôas, 2018).

In the Amazonian region, the development of an herbal medicine value chain is particularly strategic. It strengthens the local manufacturing sector through sustainable extraction and cultivation of Amazonian plant resources, leading to the creation of bioproducts via agroecological systems. This approach not only boosts the socioeconomic and environmental empowerment of local communities but also ensures they benefit from sharing rights for the use of genetic resources and associated traditional knowledge. Thus, the consolidation of a regional science, technology, and innovation system can prevent the Amazonian people from being mere commodity suppliers, promoting a diversified and sustainable economic landscape instead (PROFitos BioAM, 2021).

Brazil appears internationally among the most productive countries in bibliometric studies with a global focus based on ethnopharmacological literature (Popović et al., 2016; Yeung et al., 2020), in medicinal plant research trends (Salmerón-Manzano et al., 2020) and in pharmacology and toxicology of natural products (Chen et al., 2020). It also appears in a prominent position in bibliometric studies on medicinal plants or ethnobotanical fields in the context of Latin America and the Caribbean (Alarcon-Ruiz et al., 2023) and specifically in the context of Brazil (Ritter et al., 2015; Zago, 2018). To date, however, no bibliometric study has been found on medicinal plants and herbal medicines with a specific focus on the Amazon and with more in-depth analyzes of the network of scientific production among researchers/authors. This level of analysis allows not only to visualize the groupings of scientific production partnerships among authors, but also to identify the areas and lines of research of each of them. From our understanding, the analyzes of groups of authors are those that most reflect real partnerships, since they reduce the biases caused by sums of scientific production within institutions and countries that are not necessarily in collaboration.

This article seeks to increase the Amazon region's role in the technical and scientific advancement of herbal medicines, address the fragmentation of initiatives and elevate medicinal plants from Amazonian biodiversity as strategic assets for regional development. Understanding the distribution of technical-scientific knowledge on medicinal plants and phytotherapeutics, and visualizing research partnerships and networks, can aid in shaping a future that enhances the development of related value chains. Such insights can highlight beneficial aspects of past research and development (R&D) efforts and encourage new initiatives necessary for the progress of R&D in this economic sector. Additionally, it can facilitate innovative approaches to planning and executing R&D by leveraging existing research networks and expertise, both within Brazil and internationally, especially in areas that are currently underrepresented.

The study aims to provide a comprehensive overview of the national and international technical-scientific production related to medicinal plants and herbal medicines in the Amazon from 2002 to 2022. It encompasses (i) bibliometric analysis to identify and characterize research output, (ii) social network analysis based on graph theory to examine the structure and relationships within the research network, and (iii) Technological Readiness Level (TRL) analysis to evaluate the scientific production's maturity and the innovation system's development.

## 2 Materials and methods

Our approach encompassed several essential steps: data collection, data cleaning, bibliometric analysis, network analysis, and categorizing publications based on their Technological Readiness Level (TRL) using advanced Natural Language Processing (NLP) techniques such as Latent Dirichlet Allocation (LDA) and the Jaccard distance measure.

### 2.1 Material

For data collection, we conducted a search to retrieve academic papers published between 2002 and 2022 from Scopus database. We chose Scopus rather than other scientific databases due to its comprehensive coverage in terms of geographical regions, journals, and subject areas, and robust data integrity. The timeframe was chosen to represent the scientific outlook on the field in the last 20 years. The search query^1^ targeted academic publications that contained in the Title, Abstract, or Keywords the word “Amazon” (and variations) together with at least one of 77 descriptor terms related to medicinal plants, in both Portuguese and English. The descriptor terms were selected based on the most relevant keywords in reviews and articles about medicinal plants and herbal medicines from the last 10 years in order to include all the relevant and recent terms referring to the topic.

### 2.2 Methods

Following data extraction from Scopus on May 4th, 2023, in CSV format, we uploaded the data on the software Vantage Point for pre-processing and data cleaning—this involved removing duplicate records, and correcting countries, institutions, and authors' names, as well as refining the list of author keywords. Vantage Point is a text-mining software developed by “Search Technology Inc.” and defined as “a powerful text-mining tool for discovering knowledge in search results from patent and literature databases” (https://www.thevantagepoint.com/). Basic metrics analysis, such as annual publication records and major contributions from countries, institutions, and authors, was conducted using Microsoft Excel.

#### 2.2.1 Research communities and research topics through network analysis

To analyze major research communities and their collaborative efforts, we used co-authorship network analysis, which focuses on the relationships at the levels of countries, institutions, and authors. In this method, a node represents a country, institution, or author, and a link between two nodes indicates a co-authorship. We use Gephi, a network analysis and visualization software, to create and visualize our research communities and networks. The node size reflects the number of publications, the color represents the cluster identified by the modularity algorithm in Gephi (Blondel et al., 2008), and the link thickness indicates the strength of the partnership, based on the number of collaborative publications. Vantage Point and Microsoft Excel were also used to create and format the adjacency matrices of countries, institutions, authors, and keywords, which were later uploaded in Gephi software to visualize the co-authorship and thematic networks. Such analyses are crucial in understanding interdisciplinary collaborations and the evolution of scientific activities, as they reveal stable social ties (Jeong and Koo, 2016; Chen et al., 2020).

The analysis of the evolution of research topics was performed in two different periods, from 2002 to 2012, and from 2013 to 2022. For the network of topics, the adjacency matrix of co-occurrence of total keywords (author and indexed) was extracted from Vantage Point and uploaded in Gephi, similar to the process described above. In this case the node size represents the number of publications containing the keyword, and the edges represent the co-occurrence of both keywords in a publication.

#### 2.2.2 Technological maturity levels of research

The Technology Readiness Level (TRL) is a metric system initially used by the U.S. National Aeronautics and Space Administration (NASA), which allows to evaluate, at a given moment, the maturity level of a particular technology, as well as to compare the maturity of different types of technologies (Banke, 2010). It encompasses nine scalable levels of technology maturity, with the first being the lowest and the ninth the highest. Each economic sector presents its specificity in defining the technology levels. Strongly regulated sectors present more defined technological stages, and one only advances to the next stage with evidence fulfilled at the previous level (Velho et al., 2017). This is the case of R&D of herbal medicines, whose levels of clinical testing in humans, with new drug molecules, can only occur if non-clinical studies, *in vitro* and/or *in vivo* models, in experimental animals, have been conducted, with evaluation of the minimum potential for toxicity and observation on the occurrence of carcinogenicity, teratogenicity and mutagenicity (Brasil, 2014; Velho et al., 2017).

Our TRL classification were adapted from Velho et al. (2017), which analyzed different TRL types and exemplified the use of TRL for drug development, and also from InovafitoBRASIL (2023), which is a Brazilian platform whose motivation is to enable the production of new herbal medicines from the approximation of actors in the innovation ecosystem. Therefore, we considered at the: (i) level 1, the so-called basic researches, which identify extracts; (ii) level 2 performs isolation of substances, fractionations, chemical characterization; (iii) level 3 performs *in vitro* and *in silico* tests, included here because it is most commonly performed at this stage of research; (iv) level 4 performs *in vivo* tests with animals; (v) level 5 conducts formulation research and begins to submit patents; (vi) level 6 conducts phase I clinical tests, which aims to prove the safety of the product applied acutely in healthy individuals to define the appropriate dosage, that is, to preliminarily evaluate tolerability and pharmacokinetics; (vii) level 7, phase II clinical trials, aims to assess efficacy and side effects (safety) in a small group of sick subjects; (viii) level 8, phase III clinical trials, which aims to assess long-term efficacy and side effects involving a larger group of sick subjects and cost effectiveness; (ix) level 9, phase IV clinical trials, which aims to identify adverse effects that may occur after marketing.

To implement our TRL classification, we developed an algorithm that followed a series of steps: (1) Defining a set of keywords associated with each TRL, which served as the basis for our classification criteria (Annex 1); (2) Manual classification of 107 papers, based on a random sampling, to establish a benchmark, made by a group of experts that classified the papers into the following TRL categories: TRL1, TRL2, TRL3, TRL4-5 (grouped), and TRL6-9 (all clinical trials); (3) NLP-Based classification based on the input from step 1 and step 2, along with the content of the papers' abstracts.

Our methodology has been designed with a focus on capturing the nuances of technological maturity within academic research. One inherent limitation is the difficulty in directly capturing TRL5 activities, such as patent submissions, through academic publications, since patents are not always reflected in scholarly articles. Acknowledging this limitation, we have grouped TRL4 and TRL5 due to the understanding that TRL5 represents a turning point in the research and development (R&D) lifecycle that signifies the technological maturation of a laboratory or research group. While patents may be filed during earlier research phases (at levels 2, 3 or 4), TRL5 distinctively encapsulates the readiness for protection and commercialization of technology, which is a key indicator of both the innovation's advancement and the research team maturity. Moreover, the proposed method has not yet made it possible to separate the different phases of clinical tests (I, II, III, and IV) that correspond to TRL 6 to 9. We, therefore, chose to group them into a single category, to identify the articles that already have research that performed clinical tests indifferently from their phases, which is an important information not only to show the technological maturity, but also due to the possibility of interest from industry, which generally finances these phases of R&D.

It is important to note that all collected papers have titles, abstracts, and keywords in English, even when the original language is other than English, so they didn't require any specific handling in order to perform our analysis. We trained a Latent Dirichlet Allocation (LDA) model within a Python environment and experimented with various distance measures for TRL classification, including Hellinger (Shemyakin, 2023), Kullback-Leibler (Bigi, 2003), Jensen-Shannon (Connor et al., 2013), and Jaccard (Jiang et al., 2011). Ultimately, we opted for the Jaccard distance measure, which yielded a 22.4% error rate—demonstrating superior performance compared to other measures. By utilizing this comprehensive methodology, we aimed to establish a systematic and data-driven approach to classify research papers into relevant TRL stages. Our code is available in the online repository https://github.com/murarosilva/Profitos (Mazoni and Muraro da Silva, 2023).

## 3 Results

### 3.1 Basic metrics

Our search retrieved 1,850 publications related to medicinal plants and herbal medicines in the Amazon from Scopus platform, published between 2002 to 2022. The majority of these documents were articles, accounting for 88.5% (1,638 publications), with review articles making up 7.2% (133 publications), and the remainder being of lesser relevance. English was the predominant language for these publications, comprising 89.5% (1,697 publications), while Portuguese and Spanish represented a smaller fraction, at 6.8% (128 publications) and 2.9% (55 publications) respectively. The search also uncovered 2,292 chemical substances and 1,386 chemicals listed in the Chemical Abstracts Service (CAS). Researchers from 95 different countries and territories contributed to the entirety of the 1,850 papers.

**Figure 1:**
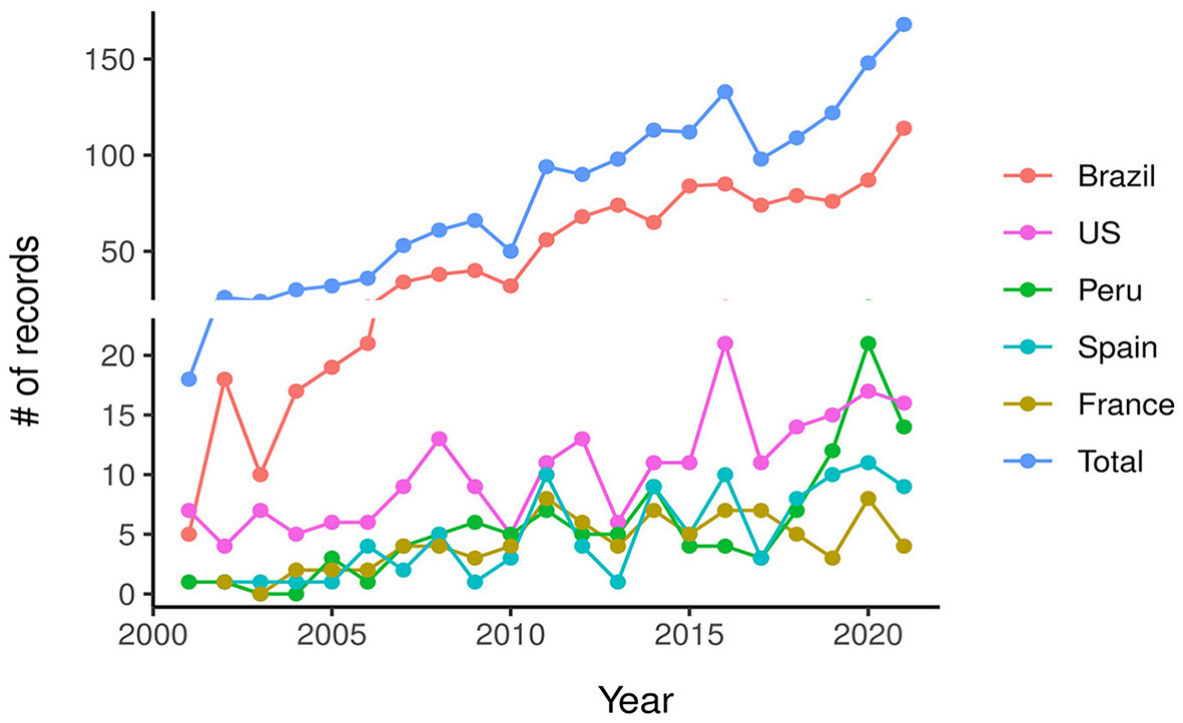
Evolution in the number of publications on medicinal plants and herbal medicines related to Amazonia for all countries and for the five most productive.

**Figure 2:**
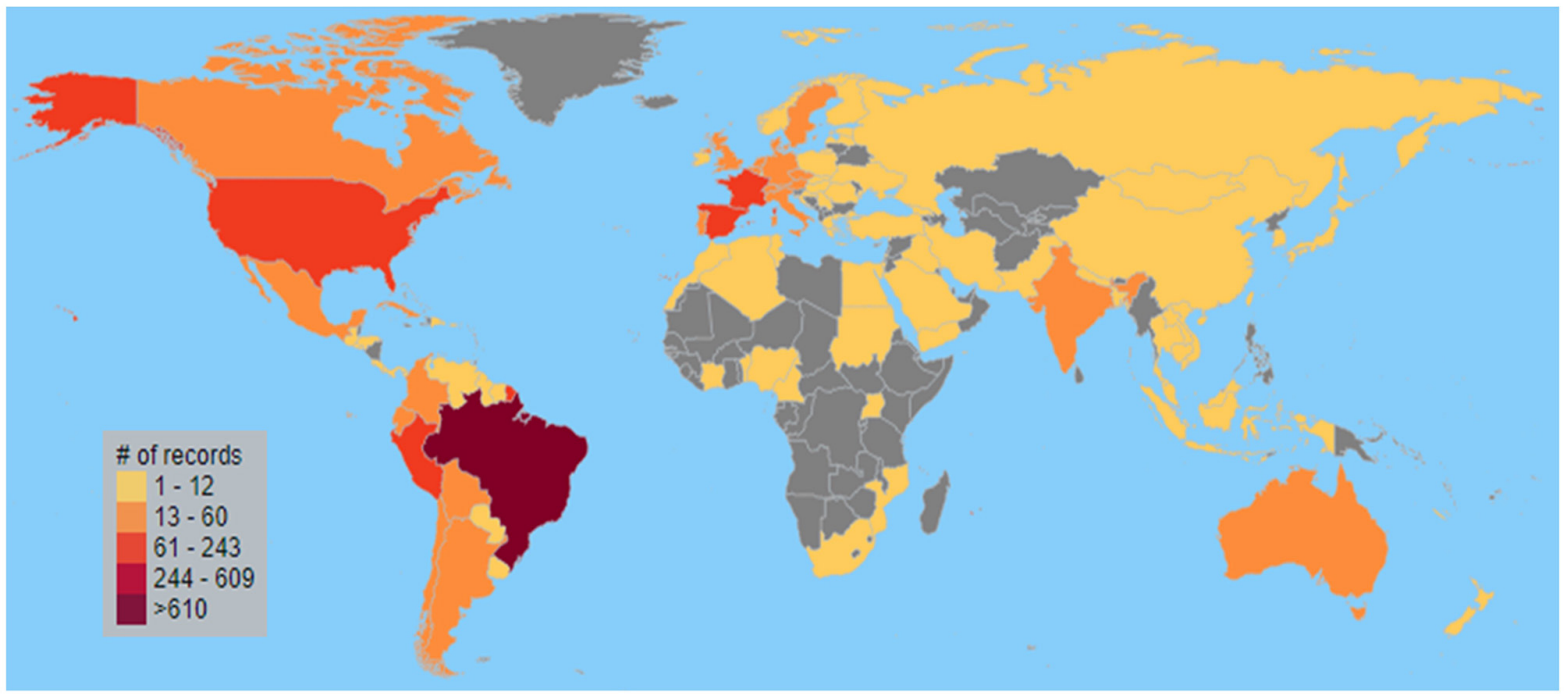
Geographical distribution of the number of scientific production on herbal medicines related to the Amazon from 2002 to 2022.

Post-2010, there was a noticeable increase in the volume of publications, with Brazil leading the surge, accounting for 65.9% of the total output (Figure 1). Among the top five publishing countries, Brazil was followed by the United States at 12.9%, Peru at 6.9%, Spain at 5.5%, and France at 5.0%, as shown in Figure 2. The number of publications from the latter four countries remained relatively stable over time.

Brazilian publications had the highest sum of citations (23,041 citations), followed by publications from the US (5,514), Spain (3,433), Peru (2,338), France (2,125) and the United Kingdom (UK) (1,622). Despite leading in both the count of published records and total citations, Brazil's citation ratio—the average number of citations per published record—was 18.9. This figure is lower than those of Spain with a ratio of 33.7, the UK at 28.5, Germany at 24.7, the United States at 23, and France at 22.8, among the most prolific countries. A similar pattern of fewer publications but higher citation ratios was observed in countries like India, Canada, the Netherlands, and Belgium.

### 3.2 Institutions

**Figure 3:**
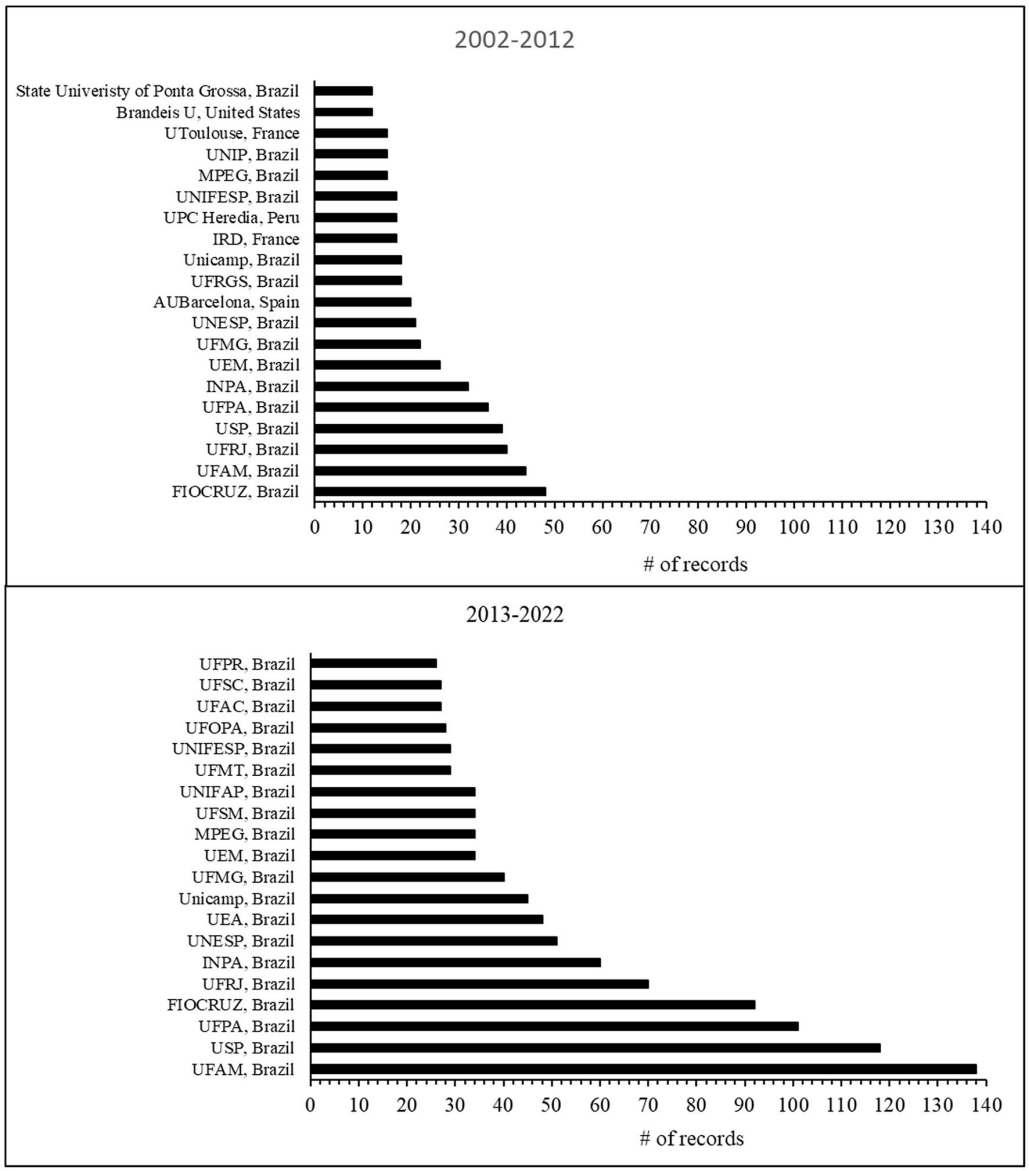
Number of publications from the 20 most productive institutions in the periods 2002–2012 (*N* = 567 records) and 2013–2022 (*N* = 1,283).

The search identified publications from 1,783 institutions. Among these, only five, all Brazilian, published over 100 papers each: Federal University of Amazonas (UFAM), University of São Paulo (USP), Oswaldo Cruz Foundation (Fiocruz), Federal University of Pará (UFPA), and Federal University of Rio de Janeiro (UFRJ). A total of 11 institutions have published 50 or more papers, 51 institutions have published at least 20, and 84 institutions have reached the 10-publication mark. During the separate analysis of two distinct periods, from 2002 to 2012 and from 2013 to 2022 as shown in Figure 3, Brazilian institutions were prominent in the list of the 20 most productive. In contrast, international institutions like the Autonomous University of Barcelona (AUBarcelona-Spain), the Institut de Recherche pour le Développement (IRD-France), Universidad Peruana Cayetano Heredia (UPCHeredia), University of Toulouse (UToulouse-France), and Brandeis University (BrandeisU-USA) made their appearance only in the earlier period from 2002 to 2012.

### 3.3 Main authors

**Figure 4:**
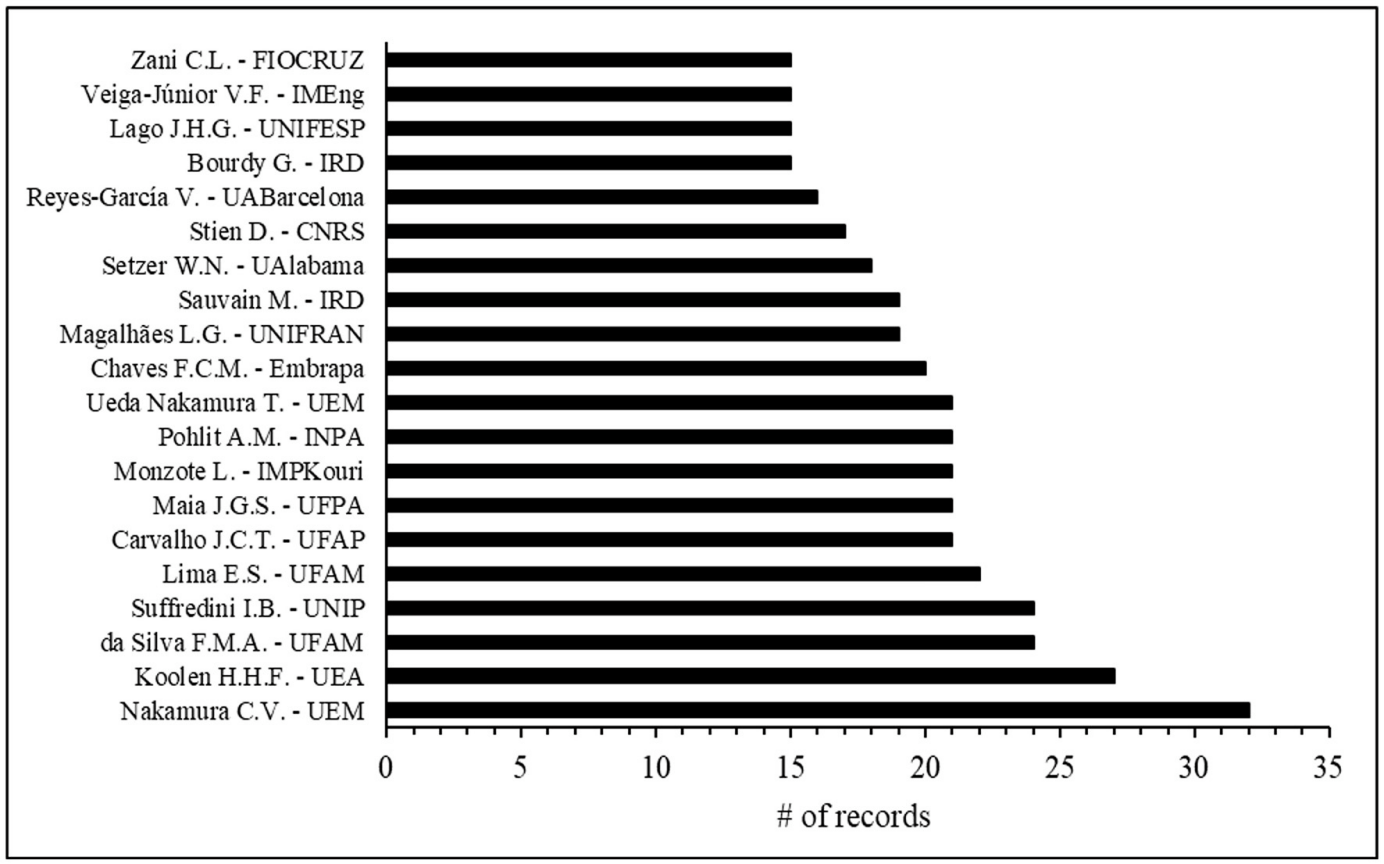
The 20 most productive authors between 2002 and 2022.

The analysis identified 8,046 authors who contributed to publications in the field. Of these authors, 277 had published five or more documents, 68 had authored 10 or more, and a select group of 11 had produced 20 or more publications. Among the 20 most prolific authors, all but five were Brazilian—the exceptions being L. Monzote from Cuba's Institute of Tropical Medicine Pedro Kouri, M. Sauvain from France's IRD, V. Reyes-Garcia from Spain's UABarcelona, D. Stien from France's CNRS, and W. Setzer from the USA's University of Alabama (UAlabama). Notably, seven of the leading authors were affiliated with Amazonian institutions, highlighting the region's significant contribution to research, despite the marked asymmetry in terms of number of researchers between the north region and other regions further to the south of Brazil in many research areas (Figure 4).

### 3.4 Research categories

**Figure 5:**
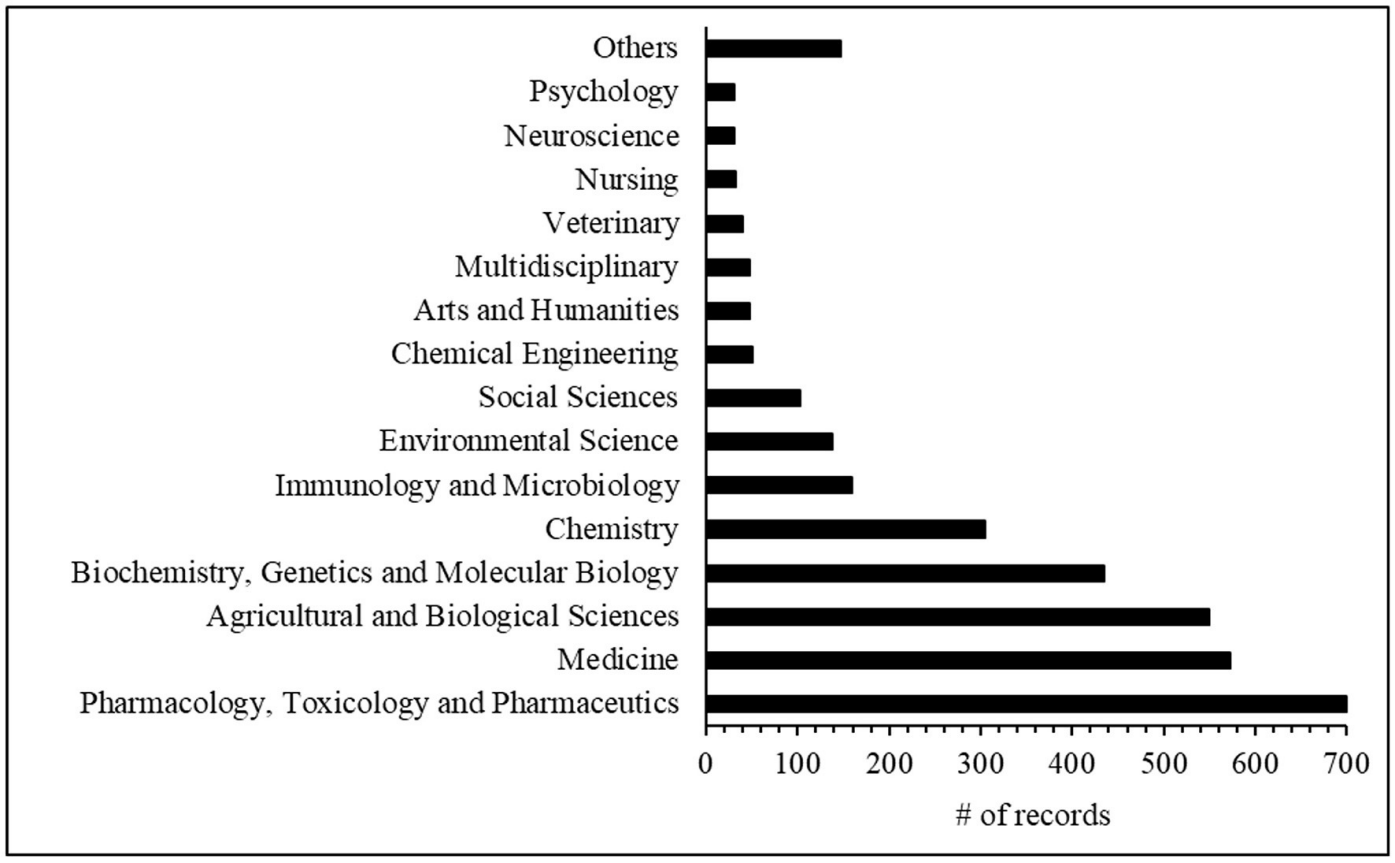
Number of publications on medicinal plants and herbal medicines related to the Amazon by research area as classified by Scopus (2002–2022).

Figure 5 shows the main research categories reflected in the publications, considering that Scopus classifies each document in more than one subject area. The five most represented research categories (with more than 300 records) included Pharmacology, Toxicology & Pharmaceutics, Medicine, Biochemistry and Chemistry, providing convergence between the terms used in the Scopus search and the capture of publications with a focus on development of herbal medicines.

### 3.5 Research communities through scientific collaboration

**Figure 6:**
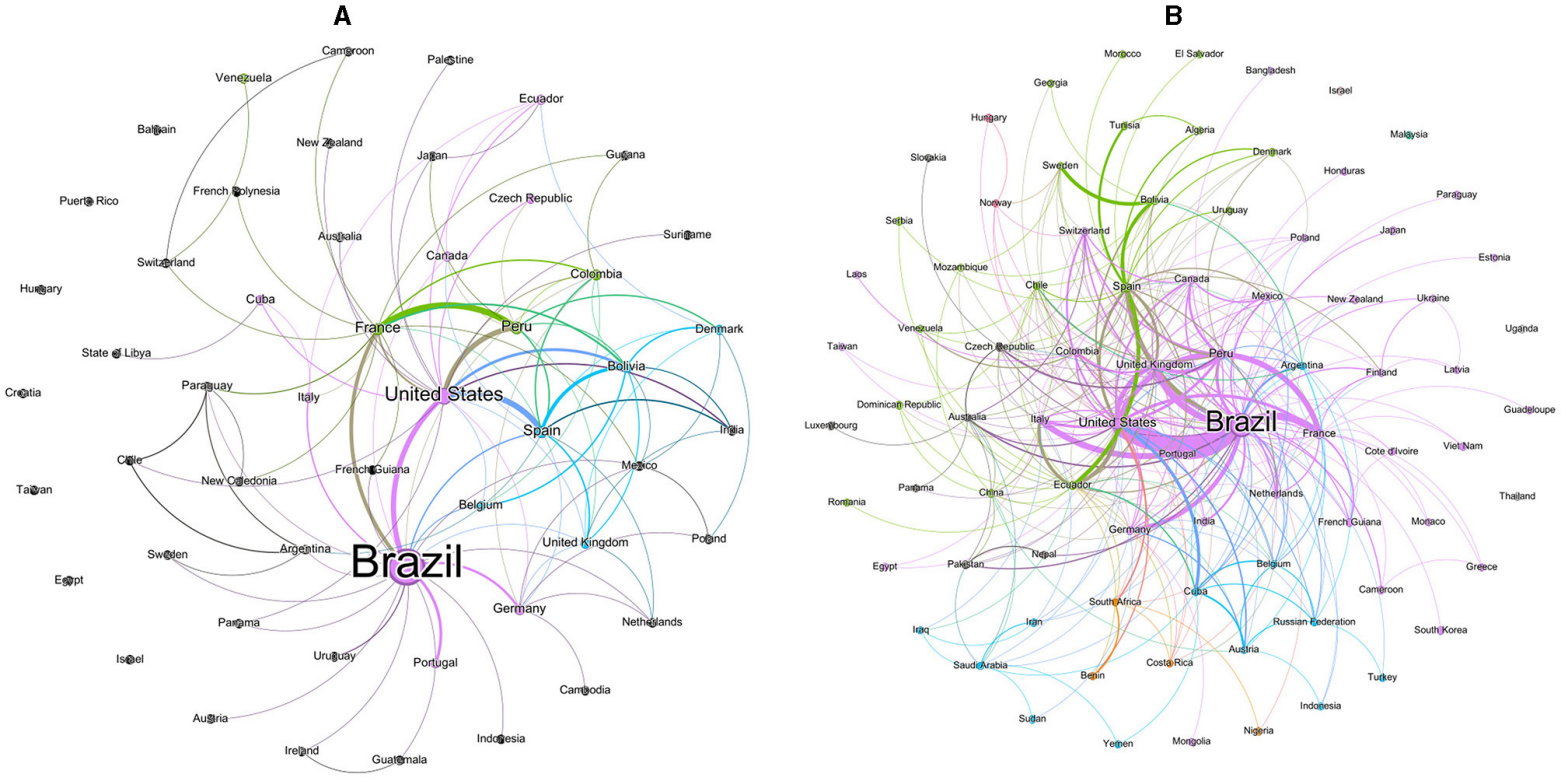
Network visualization of scientific collaboration among countries in 2002–2012 **(A)** and 2013–2022 **(B)**.

Network analysis can identify the collaborative relationships among countries, institutions, and authors, offering insights into the exchange and dialogue on specific subjects. Our analysis focused on networks of cross-country research collaborations with at least 10 publications related to medicinal plants and herbal medicines in the Amazon and were divided in two periods. The number of publications captured between 2002–2012 (567) and 2013–2022 (1,283) more than doubled (Figure 6). In the first and second periods, the same five countries stand out in the production of publications, Brazil, United States, France, Peru and Spain, with the first two leading. In the second period, there was also representation from the United Kingdom. In the first period, Brazil had partnerships mainly with the US and France and, to a lesser extent, with Portugal and Germany (Figure 6A). The US also cooperated with Spain and Peru and, to a lesser extent, with France. Spain, together with the US, cooperated with Bolivia. In the second period, partnership relationships become more complex and diversified (Figure 6B). There are two groups of more cohesive relations, one led by Brazil and the US, in partnership with France, UK, Italy, Germany, Peru, Colombia and India, and another with weaker relations between Spain, Ecuador, Bolivia and Sweden. Finally, a third group of few relations between Cuba, the US, Austria, the Russian Federation and Belgium.

**Figure 7:**
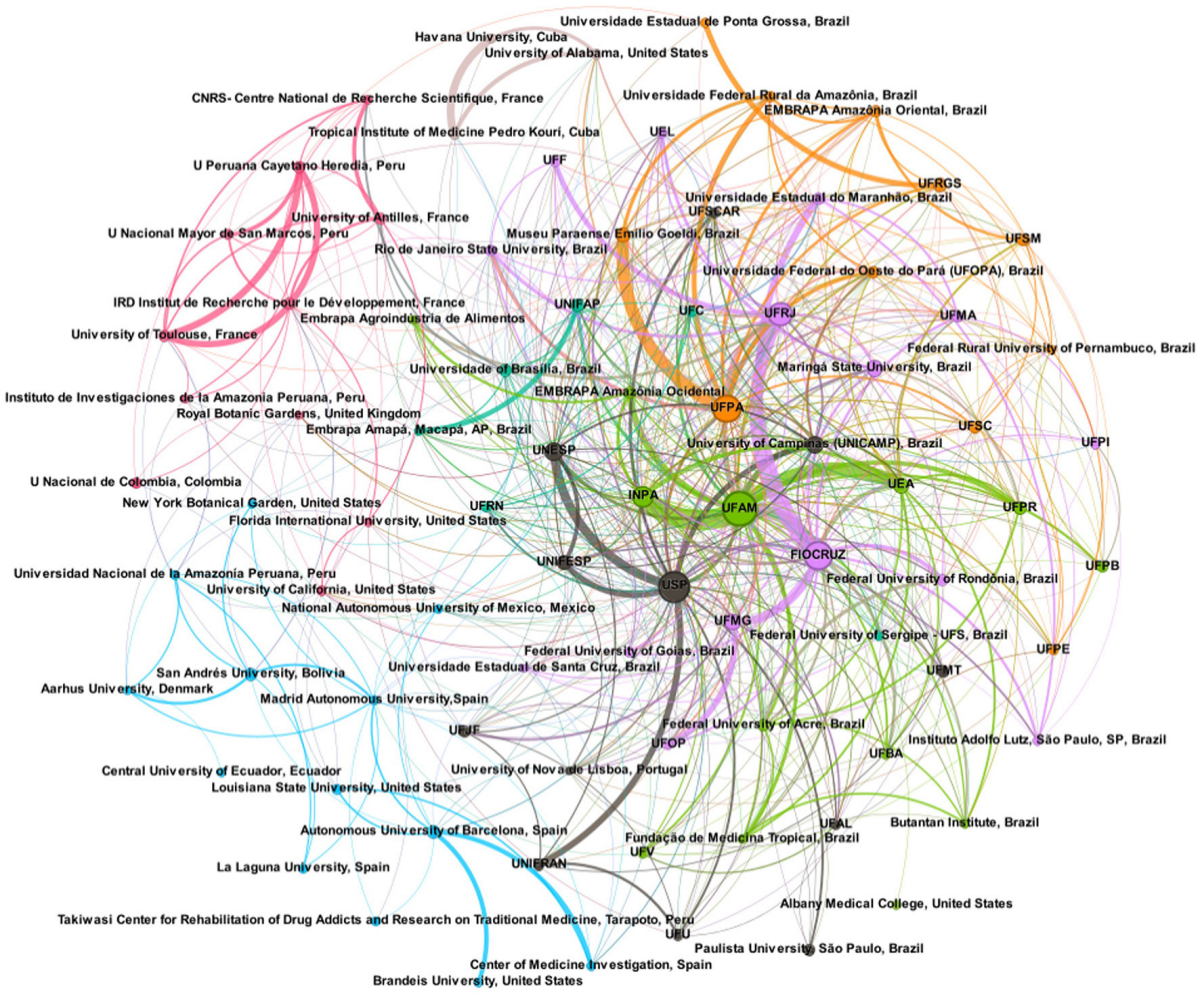
Networks visualization of core scientific collaboration among institutions with a minimum of 10 documents by institution.

The network analysis based on co-authorships among institutions revealed 84 institutions with over 10 publications, forming four major clusters predominantly composed of Brazilian institutions centralized and to the right in the network visualization, in Figure 7. Conversely, European and other South American institutions formed three smaller clusters to the left, characterized by sparser interactions.

The green cluster primarily includes institutions from the Brazilian Western Amazon. The Federal University of Amazonas (UFAM) exhibited strong collaborations with the National Institute for the Amazon Research (INPA), Amazonas State University (UEA), Embrapa Western Amazônia (Embrapa/AM), the Tropical Medicine Foundation (FMT), and the Federal University of Paraná (UFPR). The gray cluster represents institutions predominantly from São Paulo State, including the University of São Paulo (USP), Paulista University Júlio de Mesquita Filho (UNESP), Federal University of São Paulo (UNIFESP), University of Franca (UNIFRAN), and the State University of Campinas (Unicamp). Other institutions with less output include the Federal University of São Carlos (UFSCAR), Paulista University (UNIP), and the Takiwase Center for Rehabilitation of Drug Addicts and Research on Traditional Medicine in Peru (Tarapoto, Peru). The lilac cluster is geographically diverse, encompassing institutions from the Western Amazon and the Southeast and Northeast of Brazil. This cluster is led by the Oswaldo Cruz Foundation (Fiocruz), with significant ties to UFRJ, UFAM, the Federal University of Maranhão (UFMA), State University of Maranhão (UEMA), Federal University of Acre (UFAC), Federal University of Rondônia (UFRO), USP, Federal University of Minas Gerais (UFMG), and Embrapa Food Agribusiness (Embrapa/Agro). Lastly, the orange cluster consists mainly of institutions from Eastern Amazonia. The Federal University of Pará (UFPA) maintains solid collaborations with Museu Paraense Emílio Goeldi (MPEG), Federal Rural University of the Amazon (UFRA), Federal University of West of Pará (UFOPA), and UFRJ.

**Figure 8:**
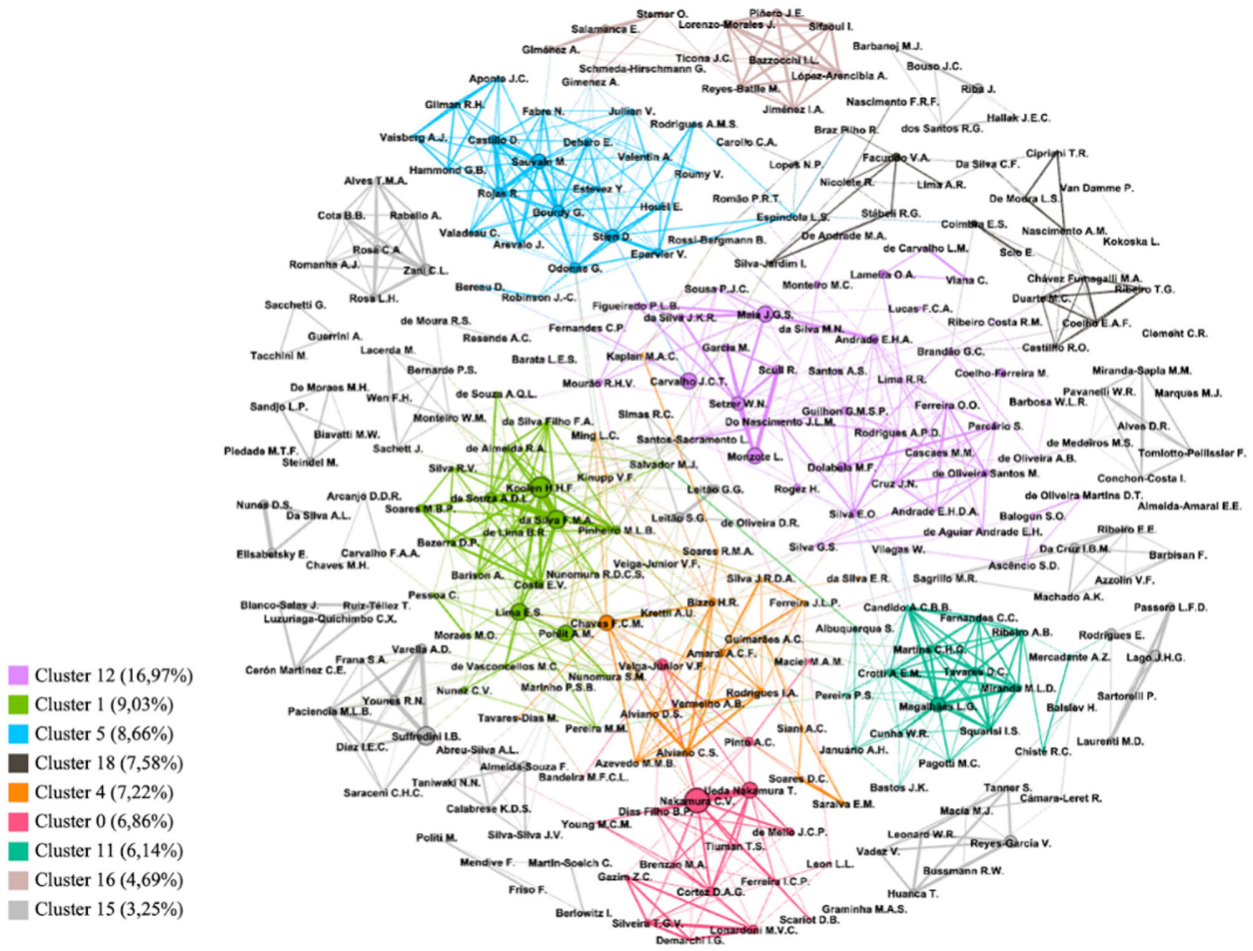
Network visualization of co-authorship among authors with a minimum of five documents per author and the percentage of the number of authors in each cluster.

The networks of co-authorship between authors were generated from 277 authors with a minimum of five publications each. This mapping revealed eight distinct clusters representing areas of focused and productive collaboration. In addition, there were other, more isolated clusters with fewer authors, indicating less productivity (Figure 8). We have detailed the key clusters below, highlighting their most influential researchers, the number of publications each has contributed to, the most frequently cited keywords, and the primary topics of research. Comprehensive details on these elements are available in the Supplemental material or upon request to the corresponding author.

a. Lilac Cluster (Number 12): This cluster includes 47 authors (17% of 277), predominantly from Brazilian institutions in the Eastern Amazon. It's led by J.C.T. Carvalho (UNIFAP), J.G.S. Maia and M.F. Donabela (UFPA), E.H.A. Andrade and M. Coelho-Ferreira (MPEG), and also includes significant contributions from Cuban institutions, with key authors like L. Manzote (IMPKouri, Cuba) and R. Scull from University of Havana (UHavana, Cuba), along with W.N. Setzer (UAlabama, USA). The number of researchers in this network is about double the second most productive group and the most productive in terms of numbers of publications (a total of 208 publications). The most frequent author keywords in this group are Amazon, medicinal plants related to plant-derived agents with antileishmanial, antimicrobial and antimalarial activities, parasitology and search for new drugs for neglected diseases (leishmaniasis, helminths, schistosomiasis, and malaria). Moreover, it can be found tests of cytotoxicity, anti-inflammatory, analgesic and anti-gastric ulcer activities, and studies with phenolic compounds, flavonoids, tannins, terpenes, volatile compounds (essential oils and aromas), and fatty compounds and dyes.
b. Green Network (Number 1): Comprising 25 authors (9%) with 106 publications from Brazilian institutions in the Western Amazon, led by H. H. F. Koolen from UEA, F. M. A da Silva, E. S. Lima, A. D. L. de Souza, M. L. B. Pinheiro and E. V. Costa from UFAM, and A. M. Pohlit e C. V. Nunez from INPA. Authors of this network have relatively stronger connections than the previous cluster, and, to a lesser extent, partnerships with the orange network (number 4). The most frequent author keywords in this cluster are Amazon, cytotoxicity, antimalarial, antileishmanial and antiplasmodial activities, and studies with flavonoid and triterpene compounds and antioxidant, anti-tumor and anti-inflammatory activities. Its main research focuses on extraction of natural products and secondary bioactive metabolites of medicinal and agronomic plants, bioprospecting and evaluating bioassays for neglected diseases.
c. Blue Cluster (Number 5): This 24-author group (9%) consists of 65 publications, including authors from French institutions, such as M. Sauvain (IRD, France), D. Stien and G. Odonne (CNRS, France), G. Bourdy and E. Deharo (UToulouse, France), and from Peruvian institutions such as D. Castillo and Rosario Rojas from UPCHeredia, and Universidad Nacional Mayor de San Marcos. It is a very isolated cluster, but with relatively strong partnerships among authors. It is noteworthy that the French researchers usually are part of the faculty of Peruvian universities. The most frequent author keywords in this group are Leishmania and related terms, medicinal plants, Peru, traditional medicine, and ethno-studies, including, Chayahuita, Ethnopharmacoly, Ethnomedicine, and Ethnobotany. The main research activities of this cluster are parasitic tropical diseases and chemistry of natural substances isolated from Amazonian biodiversity against infectious agents for neglected diseases. The ethno-studies include also research with indigenous Archaeololgy, Cosmology (Anthropology) and ethnopharmacology to select medicinal plants with antiparasitic properties used by traditional populations for pathologies principally in Bolivia, French Guyana, and Peru.
d. Black Cluster (Number 18): Composed of 21 authors (8%) with 68 publications, but their relationships are weaker, led by V. A. Fecundo from Federal University of Rondônia (UFRO), E. S. Coimbra from Federal University of Juiz de Fora (UFJF) and N. Peporine Lopes from USP. Antileishmanial activities, followed by natural products/medicinal plants and cytotoxicity are the most frequent keywords. Investigations in this cluster are focused on chemistry, mass spectrometry, phytochemical and pharmacological studies of native medicinal plants for neglected diseases, including isolation, purification and structural determination of active principles from plants, bioassays, animal experimentation and controlled study.
e. Orange Network (Number 4): It is a cluster with 20 authors (7%) and 75 publications, but which also have partnerships with the green (1) and red (0) networks. The most prominent authors are F. C. M Chaves from Embrapa Western Amazonia and authors from institutions of the Rio de Janeiro State, such as H. R Bizzo from Embrapa Food Agribusiness and other authors from UFRJ, such as C. S. Alviano and I.A. Rodrigues, and A. C. Siani from Fiocruz. The most frequent author keywords in this group are Leishmania, medicinal plants, essential oils, polyamines, arginase, nitric oxide and nerolidol compounds. Researchers are mainly focused on genetic resources and cultivation of medicinal and aromatic plants, chemistry of natural products and plant bioactives against insect pests and vectors of neglected diseases (antileishmanial, anthelmintic and antimicrobial activities, fungi, bacteria and protozoa), chromatography and mass spectrometry, standardization, isolation of substances, and structural determination.
f. Red Cluster (Number 0): With 19 authors (7%) and 83 publications, this group mainly includes South and Southeast Brazilian institutions, with remarkable leadership of C. V. Nakamura and T. U. Nakamura, both from UEM. In addition, this cluster is led by other authors from UEM, such as D.A. G. Cortez, M. V. C. Lonardoni, J. C. P. de Mello e B. P. Dias Filho, with the exception of V. F. Veiga-Júnior, currently at Military Engineering Institute (IMEng) in the Rio de Janeiro State (he was a professor at UFAM), and A. C. Pinto from UFRJ. The most frequent author keyword in this group is also related to antileishmanial activities, followed by cytotoxicity, antiprotozoal activity, medicinal plant, phytotherapy and essential oils. Its investigations focus mainly on the following topics: development of natural products with antiviral, antiprotozoal, antifungal, antibacterial and acaricide activities, controlled studies *in vivo* (animal and human cell), cytotoxicity, drug structure, isolation, development and activity mainly with the Amazon tree species of the genus Copaifera L.
g. Green-Pool Cluster (Number 11): Composed of 19 authors (6%) with 48 publications from Southeast Brazilian institutions, led specifically by L. G. Magalhães and D. C. Tavares (UNIFRAN), with C. H. G. Martins from Federal University of Uberlândia (UFU). The most frequent author keyword in this group is related to antileishmanial, trypanocidal and antibacterial activities, essential oils, cytotoxicity, and antioxidant activities. It also includes investigations on mechanism of action of natural products, especially essential oils and oleoresins, applied to health and neglected parasitic diseases, evaluation of the toxicity of organic and inorganic substances through genetic, biochemical and systemic parameters, animal experiment and controlled study.
h. Pale Pink Network (Number 16): This group comprises 34 publications authored by 13 authors (5%) from Spanish and Bolivian institutions. It is led by J. Lorenço-Morales, J. E. Piñero and A. López-Arencibia from Universidad de la Laguna (ULaguna, Spain), and E. Salamanca and A. Giménez from Universidad Mayor de San Andrés. Its investigations focus on antileishmanial and trypanocidal activities.

### 3.6 Research topics over time

We conducted an analysis of author keyword representation within the entire corpus of 1,850 records. This analysis revealed a significant presence of keywords associated with neglected tropical diseases, particularly leishmaniasis, Chagas disease, and malaria, which are predominantly caused by protozoans as seen in the discussion of author partnership clusters showed above (as detailed in Table 1). Keywords indicating *in vivo* studies with animals, such as those involving rats, and controlled tests were frequently cited. These keywords are often linked with research focusing on the cytotoxicity, efficacy, and toxicity of drugs, indicating studies in advanced stages of research and development (R&D) and the onset of clinical trials. Additionally, the aggregation of certain keywords suggested a considerable focus on the preliminary stages of R&D. This pattern of keyword distribution led us to a more detailed analysis of the R&D phases, which will be discussed in the subsequent section.

**Table 1:** Keywords cited more than 300 times in the 1,850 publications.

**# Records**	**Keywords**
1,546	Animal
1,474	Drug activity, cytotoxicity, effects, identification, mechanism, efficacy, potency, screening, structure, synthesis
1,278	Plant extract
1,121	Leishmania sp., leishmaniasis, leishmanicidal activity, antileishmanial activity & agent
1,094	Mice, inbred BALB C, muse, rats, Wistar rat, Bagg albino mouse, *in vivo* study
1,065	Human, human cell
782	Nonhuman
756	Antiprotozoal activity, amastigote, promastigote
731	Medicinal plant
708	Unclassified drug
597	Controlled study
534	Ethnobotany, ethnopharmacology, Traditional Medicine
437	Gas chromatography, mass spectrometry, high performance liquid chromatography, ultra, mass fragmentography
434	Chemistry
395	Antioxidant activity
364	Plant leaf
355	Cell culture, line, tumor, proliferation
329	Trypanocidal Agents, ctuzi, antimalarials, malaria, Plasmodium falciparum, antitrypanosomal agent
316	Male
306	IC50, Inhibitory Concentration 50

To complement the topic analysis and understand the evolution of research lines over the 20 years of bibliographic production, we analyzed author keywords added to those indexed by Scopus, with a minimum of 10 occurrences, between 2002 and 2022. Over this period, we analyzed them together (2002–2022, Figure 9) and separately in two periods (2002–2012; 2013–2022).

**Figure 9:**
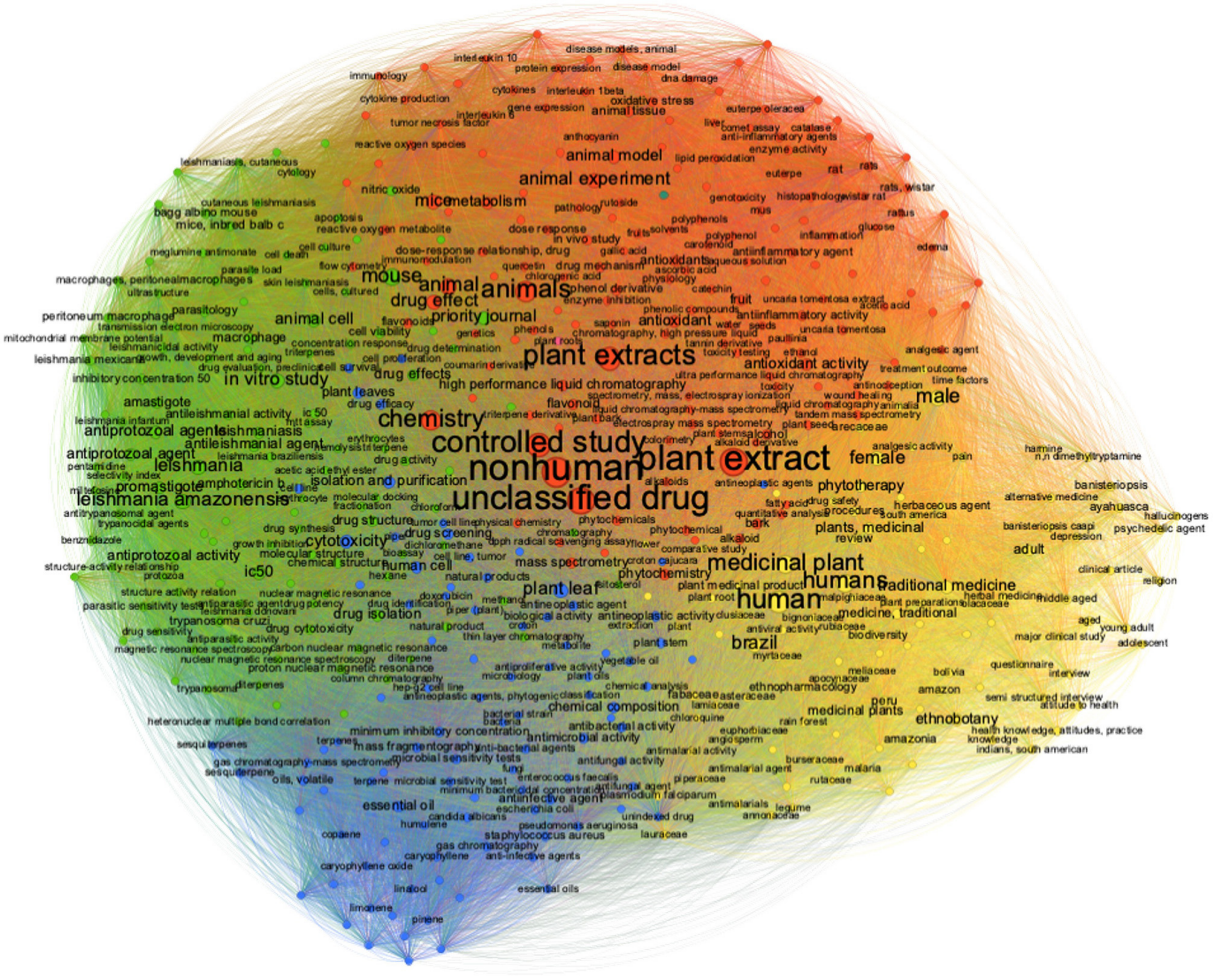
Network visualization of author and indexed keywords in 2002–2022.

For the entire period. from 2002 to 2022, it is interesting to notice that there are two well defined clusters: one headed by the term “human” (group yellow) while the other by the term “non-human” (red group) (Figure 9). In this red group there are other three keywords with almost the same level of citations: “controlled study”, “plant extracts” and “animals”. By the other hand, in the yellow group, together with “human” there are only keywords with low degree of occurrences such as the terms “female”, “male” and “Brazil”. Two other groups with less occurrences are the green, with well-defined keywords related with “Leishmania”, “antiprotozoal activities” and “IC50”, and the blue with the terms “drug screening” and “drug isolation”.

**Figure 10:**
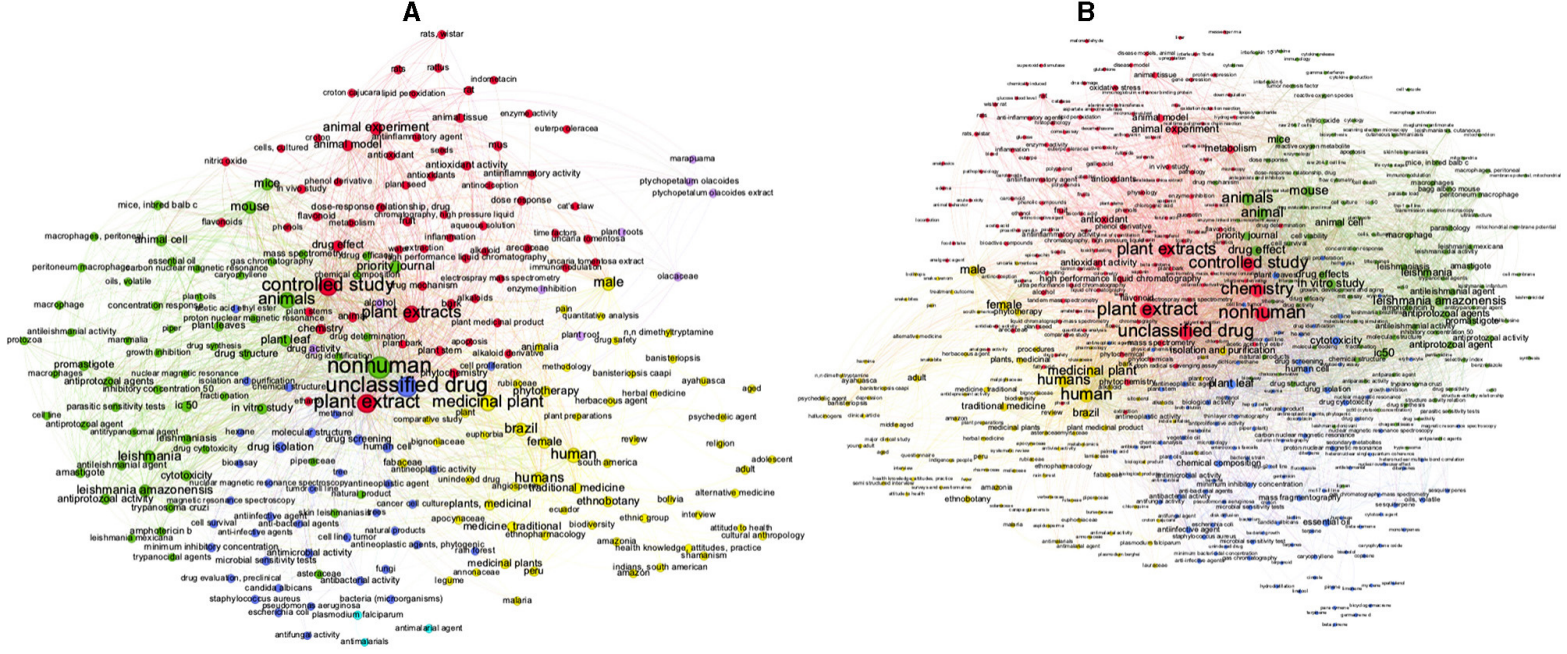
Network visualization of author and indexed keywords in 2002-2012 **(A)** and 2013-2022 **(B)**.

When we analyzed the two periods separately, it can be seen there are important differences between them (Figures 10A, B). In the first period alone (2002–2012) (Figure 10A) there are four groups with almost the same number of occurrences: one (green) with the correlations between “non-human”, “plant leaf” and “mouse”. Other group (red) with the correlations between “plant extracts”, “controlled study” and “unclassified drug”. The yellow group with the correlations between “human”, “male” and “Brazil”. And the blue group with the keywords “unclassified drug” correlated in a lower level with the terms “drug isolation” and “drug screening”. In the second period alone (2013–2022) there are very different correlations of keywords compared to the first one. In this analysis was “non-human” (red group) at almost the same level as the terms: “controlled study” and “chemistry” and in the other group “human” (yellow group) with the term's “female” “male” and “Brazil”.

### 3.7 Technology Readiness Level (TRL)

The figure below provides a detailing of the documents and their corresponding percentages across different Technology Readiness Levels (TRLs), classified by our NLP algorithm:

- TRL 1: Comprising only 26 documents, about 1% of the total, TRL 1 represents the foundational stage of research, focusing on identifying extracts and fundamental principles. The minimal representation in this category suggests a research emphasis on more advanced technology development stages rather than basic studies.
- TRL 2: This level, constituting the largest category, encompasses 834 documents, roughly 45% of the total. TRL 2 is characterized by the isolation of substances and fractionation techniques, indicating a significant concentration on the initial stages of technology development and exploration of diverse chemical compounds.
- TRL 3: It encompassed 83 documents, comprising approximately 4% of the total. This level involves *in vitro* and *in silico* tests to evaluate the properties and effects of the technology. The relatively smaller percentage suggests that there were some studies focusing on *in vitro* and *in silico* testing, they may not be the primary focus of the research.
- TRL 4-5: Encompassing 464 documents, about 25% of the total, TRL 4-5 marks the shift from laboratory-based testing to *in vivo* animal testing, including formulation research and patents. The substantial number of documents in this category indicates an emphasis on progressing technology toward real-world applications and potential commercialization.
- TRL 6-9 (Clinical Trials): The last category, TRL 6-9, comprises 442 documents, accounting for around 24% of the total. This range covers various phases of clinical trials, including Phases I to IV, but the method does not allow distinguishing them and the perception through reading some of these articles brings low expectations of publications that address tests in phases III and IV. The presence of a substantial number of documents in this category indicates a significant emphasis on evaluating the technology in clinical settings, assessing efficacy, safety, and post-marketing surveillance.

**Figure 12** shows how is the distribution of TRLs for the 5 most productive countries including Brazil, United States, Peru, Spain and France. Except for Brazil and France, all the three other countries show a higher proportion of papers classified on TRL 6-9 than the average. On the other hand, Brazil and France show higher proportions of papers than the average on TRL 2 and TRL 4-5, although Brazil has much more publications.

## 4 Discussion

The bibliometric search related to medicinal plants and herbal medicines and the Amazon prominently featured Brazil, its institutions, and authors. Post-2010, Brazil's research output significantly surpassed that of other productive countries like the USA, Peru, Spain, and France. Predominantly, studies were concentrated in Pharmacology, Toxicology, Pharmaceuticals, Medicine, and Biochemistry, showcasing advanced scientific maturity in the R&D chain's middle and later stages. Despite the small participation of other Amazonian countries besides Brazil, our results were to some extent expected due to the Amazon-centric focus of our study. Internationally, Brazil has markedly progressed in scientific production on these topics. From being ranked in fifth in 2000 to third in 2018, Brazil advanced in ethnopharmacological literature reviews (Yeung et al., 2020). Additionally, Brazil ranked fourth in medicinal plant research trends (Salmerón-Manzano et al., 2020) and third in pharmacology and toxicology of natural products (Chen et al., 2020). In these studies, Brazil consistently appeared alongside countries like China, India, the USA, Iran, and Germany, with Brazilian institutions like USP, Federal University of Paraiba (UFPB), UNESP, and UFRJ being among the most productive. In the context of Latin America and the Caribbean, a bibliometric study on medicinal plants highlighted Brazil as the most productive, reaching 64.4% of total publications, followed by Mexico and Argentina, and Brazil contributed with six of the 10 most cited publications (Alarcon-Ruiz et al., 2023). However, in another bibliometric study on natural products for cancer from 2008 to 2020, Chen et al. (2021) showed that Brazil ranked ninth in numbers of articles, with its institutions not featuring among the top 10, which was dominated by the USA. Notably, the Brazilian palm species *Euterpe oleracea* Mart. (açaí), a Brazilian palm tree species highly consumed in nutritional drinks and with medicinal properties, constituted one of the two largest early research hotspots (Chen et al., 2021).

The scientometric analysis carried out by Zago (2018) between 1991 and 2013, which used only the boolean terms “medicinal plant” or “phytotherapy” and “Brazil”, showed a slightly different pattern, with India coming first, followed by Brazil, China, USA, and Germany (the three intermediate countries presented similar numbers of articles). The number of articles captured was much higher than ours, since their search was not restricted to a specific biome/region and focused on specific plant species. A similar ongoing study^2^ that used as search terms in Scopus the scientific names of 47 species of potential medicinal plants with natural phytogeographical distribution in the Amazon and the same 77 boolean terms used here found that Brazil and India comprised half of the total number of publication records, followed by the USA, Nigeria, and China. Our hypothesis for the presence of high scientific production of India and Nigeria is the restriction related to plant species and not regions or biomes. In both cases, within the most studied species are those that are worldwide cultivated plant species and with traditionally proven therapeutic effects. In this sense, we recommend the use of species names for bibliometric searches to complement the knowledge of the scientific networks about medicinal plants and herbal medicines in a given geographic area or biome, since the plants may spread beyond these regions. Whereas 1,850 publications were captured in this article, our study of 47 Amazonian species captured 4,967 and resulted in a broader knowledge about Brazilian networks with a medicinal focus, i.e., more research topics beyond neglected tropical diseases.

We observed limited collaboration between Brazilian institutions and those from other countries, including neighboring Amazonian nations. The apparent discrepancy between the institutional (Figure 7) and country network (Figure 6), in which Brazil has strong interactions with the USA, Spain, Germany, Italy and Portugal, is due to the partnerships among countries as a consequence of publications from several institutions that have < 10 publications. Salmerón-Manzano et al. (2020) and Alarcon-Ruiz et al. (2023) showed that Brazil positioned itself as a leader in collaboration networks among countries, with strong partnerships with USA, European and Latin American countries. Our understanding is that analyzes among institutions and authors reflect much of the true relationships of scientific networks. Unfortunately, a few articles discuss networks at these levels. Chen et al. (2021) noticed similar patterns in relation to the analysis of international collaboration networks. The USA and China, countries with the largest production, have cooperated closely, but whereas the USA cooperated mainly with Italy, Australia, Germany, Spain, Taiwan, South Korea and India, China presented close relationships with Canada, France and Singapore. On the other hand, weak cooperation was observed among institutions from the USA and China. Notably, the Chinese Academy of Sciences and the US National Institutes of Health both played an important role in natural products research for cancer, but they did not cooperate with each other. The networks of non-Brazilian institutions followed the same pattern of incipient international collaborations, except a network of French institutions that had some degree of partnerships with Peruvian institutions and another network of Spain with some dispersed partnerships, mainly with South American institutions. The USA appeared to have a few institutions, so we can suppose that this country's scientific production on medicinal plants and herbal medicines related to the Amazon must be carried out by institutions that do not reach 10 publications.

The author network showed eight main clusters with more concise and productive cooperation, predominantly Brazilian, with four clusters led by authors from Amazonian institutions and two by those from South and Southeast Brazil. Author keyword of articles published in all eight author's clusters revealed a focus on neglected tropical diseases, mainly antimalarial, antileishmanial, antitrypanosomal, antiprotozoal and antiplasmodial activities. The same occurred when considering keywords from the total set of publications (leishmaniasis, *in vivo* and human studies). The study by Zago (2018) with Brazilian medicinal plants showed a similar trend: the active ingredients were tested to verify antiparasitic properties (46.8% of the records). Several diseases, including schistosomiasis, Chagas disease and malaria, also seem to contribute to increasing the amount of research aimed at discovering new phytomedicines related to the Amazon.

The analysis of total keywords (author plus indexed) allows to see the migration of the keywords that is directly linked to the migration in the focus of research about plants. In the period of 2002–2012 (Figure 10A), scientist could publish articles with *in vitro* plant extracts activities. But in the period of 2013–2022 (Figure 10B), it decreased, and instead it grew up the chemical identification of substances, represented in the terms: “chemical composition”, “proton nuclear magnetic resonance” and “mass”. As the scientific journals became more exigent, the scientific community had to adapt, and it also reflects on the terms present in Figure 9 which shows the whole period. So, the terms: “chemical composition”, “proton nuclear magnetic resonance” and “mass spectrometry” are also in the whole period (2002–2022, Figure 9). The other two groups, green and blue (Figures 10A, B), increased in keyword complexity. The green group in the more recent period has a greater representation of keywords related to Leishmania, showing an increase in searches related to the topic. The blue group, less representative in terms of more frequent keywords, was the one that differed most between the two periods with additions of new words in the second period that define new lines of research.

**Figure 11:**
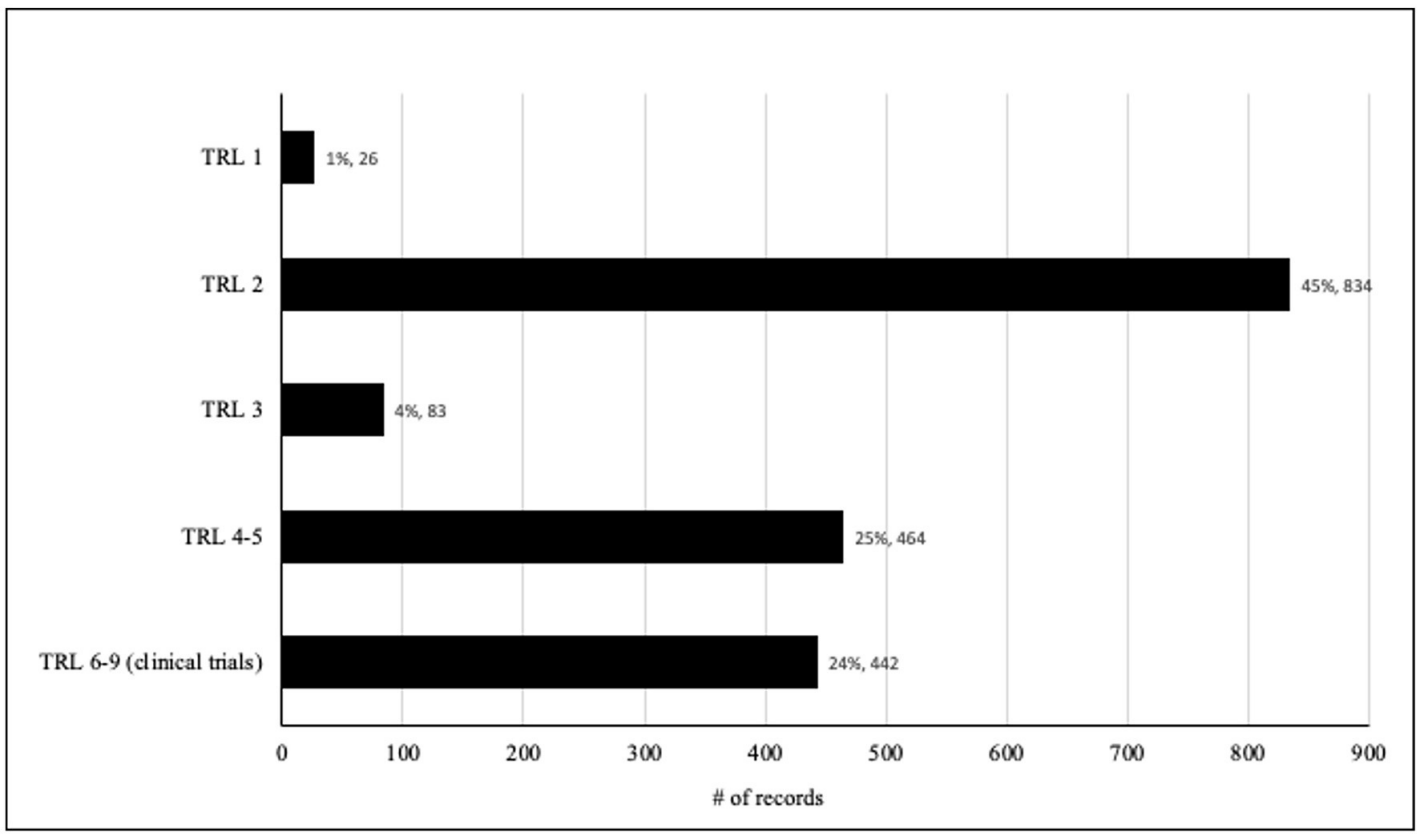
TRL classification of documents based on NLP algorithm.

**Figure 12:**
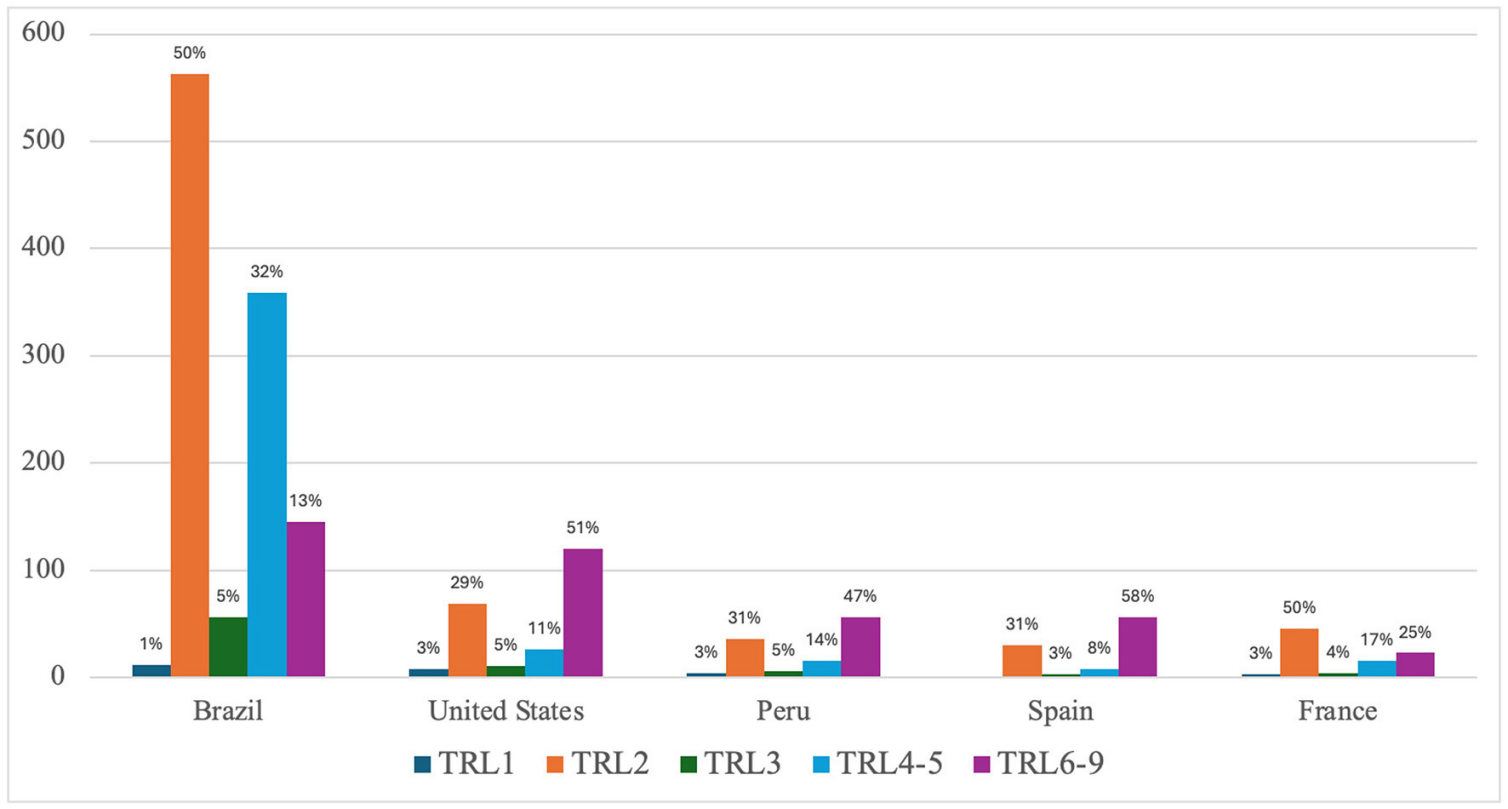
TRL classification of documents for most productive countries.

Overall, the distribution of records across different TRLs reflects a comprehensive research approach, spanning multiple stages of technological development (Figure 11). The higher proportions in TRL 2 and TRL 4-5 indicated a focus on the early development, technology formulation and the transition from laboratory-based testing to *in vivo* testing with animals. Moreover, the presence of records in TRL 6-9 indicated a significant interest in clinical evaluation and real-world applications toward potential commercialization. Regarding TRL distribution for different countries (Figure 12), the higher proportion of papers from United States, Peru and Spain on TRL 6-9 among the others TRLs suggest an interest of those countries on advancing the technology toward real-world applications and medicinal potential innovation. It is important to note that articles that analyzed responses from the population in the use of medicinal plants or herbal medicines were considered as clinical trials (TRL6-9), that is, based on the evaluation of traditional use or in response to prescription in the Brazilian Unified Health System (Sistema Único de Saúde, SUS). These studies can be identified in the keyword analysis in light green grouping (number 3) (Figure 6). Indeed, in the North of Brazil there are several studies focusing on communities that use traditional medicine to treat leishmaniosis and malaria and prevent associated symptoms (e.g., Bleil et al., 2021). We categorized clinical tests in the “broad” sense, in addition to controlled clinical tests carried out in laboratories or hospitals. We conclude that the different approaches would converge in their results, reinforcing the quality of the new proposed method of using artificial intelligence in the perception/categorization of studies in the different TRLs.

There is an important issue about the pharmaceutical industry's interest in producing medicines related to neglected diseases. It is common in articles and lectures of specialists to address the lack of effective and safe medicines aimed at treating these diseases that affect a large portion of the population with scarce financial resources and poor health care (Weng et al., 2018; Bleil et al., 2021). However, the study by Garrido-Cardenas et al. (2021) assessed the transfer of parasitology research to patents worldwide from 1996 to 2019. Twelve institutions with the highest number of parasitology publications cited in international patents are from the USA, the UK, and France (three each), and Brazil, Switzerland, and Australia (one each). Up to 15% of the articles have an industrial impact in this area, and infections known as neglected tropical diseases are the ones that are attracting the most interest from industry, including malaria, Schistosomiasis, Leishmaniasis, Visceral Leishmaniasis, and Chagas disease. As a result, they pointed out that the interest in neglected tropical diseases is not only a political, medical or social issue, but also it comes from an industry perspective, and concluded that Brazil occupies an important place in the interest list to the industry besides the US and the UK.

The STERN REPORT (2006), currently corroborated by studies of high scientific significance summarized in the 2022 Report of the Intergovernmental Panel on Climate Change (IPCC, 2022), pointed out that diseases from warmer tropical regions will spread to higher latitude regions. A new global perspective on industry investment in the R&D and commercialization of medicines for neglected diseases can also benefit tropical countries. As shown here, Brazil and its Amazonian institutions have been showing leadership in scientific production (see also Bai et al., 2016; Sampaio et al., 2017; Melo et al., 2022) and Fiocruz appears among the most important scientific institutions, an authentic reference in Parasitology and Public Health research in Latin America (Garrido-Cardenas et al., 2021). However, as showed by Melo et al. (2023) in a scientometric study from 2004 to 2020, although Brazil is largely responsible for the occurrence of neglected tropical diseases in Latin America, research funding by the Ministry of Health and its partners in Brazil on the subject does not meet the population's health needs. Most funding topics involved dengue, leishmaniasis and tuberculosis, but was lacking for Chagas disease, schistosomiasis, malaria among other diseases with a high prevalence, burden, or mortality rates in Brazil, revealing stagnation over the years.

## 5 Conclusions

Our results showed that most of the scientific production on medicinal plants and herbal medicines related to the Amazon came from Brazilian institutions and a significant number of records focused on neglected diseases. If there is a window of opportunity and industrial competitive advantage for this country in the medicine sector, it is potentially related to biodiversity, sociobiodiversity and bioeconomy, that is, in the innovation, production and use of phytomedicines in a sustainable way together with the promotion of health system and the recognizing of the traditional knowledge (ABIFINA, 2021). We argued that neglected diseases are a burden for decades in tropical countries. And they probably will also be the focus of the health systems of non-tropical countries, as result of the climate changes and likely industry's greater interest in investing in this sector. Brazil owns a multitude of compounds provided by its biodiversity and medicinal plants because of the immeasurable traditional knowledge of traditional communities and indigenous peoples. This may result in a greater probability of R&D success in obtaining positive therapeutic effects with low expectation of toxicity. Thus, these findings demonstrate the need to strengthen the health research system in partnership with the evolving medicine industry, benefiting from the scientific knowledge networks already implemented and focused on neglected diseases, which are so relevant for this country. This need to bring the industrial R&D sector closer to the R&D carried out by science and technology institutions must be reinforced in Brazil, since this country appears to be relatively less productive in the final stages of herbal medicine R&D (involving clinical tests) when compared to the other countries analyzed.

The recognition of the knowledge of traditional and indigenous peoples, in areas related to the medicinal properties of plants, is deeply linked to the value and use of biodiversity for human health. The importance of preserving this knowledge, therefore, can play a fundamental role in conserving biodiversity, meeting the Sustainable Development Goals (SDGs) and the goals defined in the Paris Climate Agreement. Translating this knowledge into publications, which can prove traditional use in a safe and therapeutically effective way, is a very important step toward their use by health agencies or similar bodies to consider them in official pharmacopoeial monographs as traditional herbal products. With this, the market is supported in facilitating regulation and collective public health mechanisms can use this legal information to produce and dispense herbal medicines safely, as occurs in Farmácias Vivas, a Brazilian program included in the Unified Health System (SUS).

## Data availability statement

Publicly available datasets were analyzed in this study. This data can be found here: https://github.com/murarosilva/Profitos.

## Ethics statement

Written informed consent was not obtained from the individual(s) for the publication of any potentially identifiable images or data included in this article because we analyzed bibliometric data, and the names of authors were identified.

## Author contributions

NL-C: Conceptualization, Data curation, Formal analysis, Funding acquisition, Investigation, Methodology, Project administration, Resources, Supervision, Validation, Visualization, Writing – original draft, Writing – review & editing. VM: Conceptualization, Data curation, Formal analysis, Investigation, Methodology, Project administration, Resources, Software, Supervision, Validation, Visualization, Writing – original draft, Writing – review & editing. HEMN: Conceptualization, Data curation, Formal analysis, Funding acquisition, Investigation, Methodology, Resources, Validation, Visualization, Writing – original draft, Writing – review & editing. AM: Data curation, Formal analysis, Investigation, Methodology, Resources, Software, Visualization, Writing – review & editing. CVN: Formal analysis, Methodology, Writing – review & editing. MBMB: Funding acquisition, Resources, Supervision, Writing – review & editing.

## Footnotes

^1^TITLE-ABS-KEY (“phytomedicine” OR “fitomedicamento^*^” OR “phytotherapic” OR “fitoterapico” OR “herbal medicine” OR “medicamento fitoterpico” OR “produto tradicional fitoterapico” OR “traditional herbal product” OR “medicinal tea” OR “herbal tea” OR “cha medicina^*^” OR “drug plant” OR “herbal drug” OR “botanical drug” OR “plant drug” OR “droga botnica” OR “droga vegetal” OR “herbal remedie” OR “medicinal herb” OR “medicamento herbal” OR “healing plant” OR “planta curativa” OR “medicinal and aromatic plants” OR “medicinal and aromatic plant” OR “medicinal plant” OR “planta medicinal” OR “natural drug^*^” OR “natural product” OR “produto natural” OR “pharmaceutic^*^ plant^*^” OR “phytopharmaceutic^*^” OR “fitofarmaceutic^*^” OR “antibacterial plant” OR “planta antibacteriana” OR “antifungal plant” OR “planta antifung^*^” OR “antiviral plant” OR “planta antiviral” OR “nanoherbal drug” OR “stimulant plant” OR “planta estimulante” OR “plant extract” OR “extrato vegetal” OR “plant derivative” OR “derivado vegetal” OR “active plant pharmaceutical” OR “insumo farmacutico ativo vegetal” OR “secondary metabolite” OR “metablito secundrio” OR “complementary and alternative medicine” OR “medicina complementar e alternativa” OR “complementary medicine” OR “medicina complementar” OR “alternative medicine” OR “medicine alternative” OR “natural medicine” OR “medicina natural” OR “traditional medicine” OR “medicina tradicional” OR “herbal therapy” OR “terapia herbal” OR “phytotherapy” OR “fitoterapia” OR “herbalism^*^” OR “ethnobotany” OR “etnobotanica” OR “ethnomedic^*^” OR “etnomedicina” OR “ethnopharmac^*^” OR “etnofarmac^*^” OR “pharmacopeia” OR “farmacopeia” OR “pharmacognosy” OR “farmacognosia”) AND TITLE-ABS-KEY (“Amazon^*^”) AND PUBYEAR > 2001 AND PUBYEAR < 2023.

^2^Lepsch-Cunha, N., Nascimento, H. E. M., Muraro, V., and Bonacelli, M. B. M. (2024). Production of technical-scientific knowledge about amazon medicinal plants with potential for the development of phytotherapy (Manuscript in preparation).
