# Supplementary material for: Technical-scientific production and knowledge networks about medicinal plants and herbal medicines in the Amazon
Source: Front Res Metr Anal. 2024 Jun 12;9:1396472. doi: 10.3389/frma.2024.1396472 (PMC11199724; doi:10.3389/frma.2024.1396472)
Supplement: Supplementary file 1 [file Table_1.DOCX]

**Annex 1**

Keywords related to each Technology Readiness Level (TRL)

| **TRL 1** | **TRL 2** | **TRL 3** | **TRL 4-5** | **TRL 6-9 (clinical trials)** |
| --- | --- | --- | --- | --- |
| - alcohol - bark - domestication - drug screening - flower - fruit - inbreeding - latex - leaf - oil - plant extract - plant stem - sap - Secondary metabolites - seed - traditional knowledge | - alkaloid - carbon nuclear magnetic resonance - chemical composition - Chromatography, High Pressure Liquid - drug isolation - drug mechanism - drug structure - drug synthesis - electrospray mass spectrometry - flavonoid* - fractionation - gas chromatography - high performance liquid chromatography - HPLC-UV - isolation and purification - LC-MS - mass fragmentography - mass spectrometry - Molecular Structure - monoterpene - phenol derivative - Phenols - Polyphenols - proton nuclear magnetic resonance - Secondary metabolites - sesquiterpene - substance isolation - tandem mass spectrometry - Triterpene - Volatile oil | - amastigote - Amphotericin - animal cell - animal tissue - Anti-Bacterial Agents - Anti-Infective Agents - Anti-Inflammatory Agents - antifungal activity - antiinfective agent - antiinflammatory agent - antimalarial agent - antimicrobial activity - antineoplastic activity - antineoplastic agent - antioxidant - Antiprotozoal Agents - antitrypanosomal agent - apoptosis - biological activity - cancer - cell culture - cell line - Cell Line, Tumor - cell proliferation - cell survival - cell viability - concentration response - Cytotoxicity - Disk Diffusion Antimicrobial Tests - Dose-Response Relationship, Drug - drug activity - drug efficacy - enzyme activity - Enzyme inhibition - Escherichia coli - gastrointestinal disease - growth inhibition - growth, development and aging - IC 50 - IC50 - in silico - in vitro - inflammation - Inhibitory Concentration 50 - Leishmania - macrophage - Macrophages, Peritoneal - Malaria - mic - minimum inhibitory concentration - Microbial Sensitivity Tests - micronucleus test - nitric oxide - nonhuman - oxidative stress - Parasitic Sensitivity Tests - parasitology - peritoneum macrophage - Plasmodium falciparum - preclinical study - promastigote - Staphylococcus aureus - Trypanocidal Agents - Trypanosoma cruzi | - Amphotericin - Dose-Response Relationship, Drug - nitric oxide - drug efficacy - antineoplastic agent - cell viability - antineoplastic activity - concentration response - Macrophages, Peritoneal - inflammation - cell proliferation - apoptosis - Anti-Inflammatory Agents - growth inhibition - growth, development and aging - gastrointestinal disease - preclinical study - controlled study - Animals - mouse - Mice - animal experiment - adult - rat - In vivo - aged - Mus - Wistar - middle aged - Drug Evaluation, Preclinical - hot plate test - forced swimming test - Pre-clinical toxicology evaluation - Preclinical | - Dose-Response Relationship, Drug - major clinical study - clinical trial - clinical assessment - clinical evaluation - clinical feature - clinical outcome - clinical trial (topic - clinical competence - clinical practice - clinical protocol - Clinical studies - phase 2 clinical trial - phase 3 clinical trial - Clinical investigation - clinical observation - clinical research - Clinical routine - Clinical Trials - phase 1 clinical trial - phase 2 clinical trial |
